# Reassessing the Impact of Smoking on Preeclampsia/Eclampsia: Are There Age and Racial Differences?

**DOI:** 10.1371/journal.pone.0106446

**Published:** 2014-10-22

**Authors:** Jen Jen Chang, Jerome F. Strauss, Jon P. Deshazo, Fidelma B. Rigby, David P. Chelmow, George A. Macones

**Affiliations:** 1 Department of Epidemiology, College for Public Health and Social Justice, Saint Louis University, St. Louis, Missouri, United States of America; 2 VCU Medical Center, School of Medicine, Virginia Commonwealth University, Richmond, Virginia, United States of America; 3 Department of Health Administration, School of Allied Health Professions, Virginia Commonwealth University, Richmond, Virginia, United States of America; 4 Department of Obstetrics and Gynecology, Virginia Commonwealth University, Richmond, Virginia, United States of America; 5 Department of Obstetrics and Gynecology, Washington University, St. Louis, Missouri, United States of America; Medical Faculty, Otto-von-Guericke University Magdeburg, Medical Faculty, Germany

## Abstract

**Objective:**

To investigate the association between cigarette use during pregnancy and pregnancy-induced hypertension/preeclampsia/eclampsia (PIH) by maternal race/ethnicity and age.

**Methods:**

This retrospective cohort study was based on the U.S. 2010 natality data. Our study sample included U.S. women who delivered singleton pregnancies between 20 and 44 weeks of gestation without major fetal anomalies in 2010 (n = 3,113,164). Multivariate logistic regression models were fit to estimate crude and adjusted odds ratios and the corresponding 95% confidence intervals.

**Results:**

We observed that the association between maternal smoking and PIH varied by maternal race/ethnicity and age. Compared with non-smokers, reduced odds of PIH among pregnant smokers was only evident for non-Hispanic white and non-Hispanic American Indian women aged less than 35 years. Non-Hispanic Asian/Pacific Islander women who smoked during pregnancy had increased odds of PIH regardless of maternal age. Non-Hispanic white and non-Hispanic black women 35 years or older who smoked during pregnancy also had increased odds of PIH.

**Conclusion:**

Our study findings suggest important differences by maternal race/ethnicity and age in the association between cigarette use during pregnancy and PIH. More research is needed to establish the biologic and social mechanisms that might explain the variations with maternal age and race/ethnicity that were observed in our study.

## Introduction

Preeclampsia is associated with significant pregnancy-related morbidity and mortality, [1]. The etiology of preeclampsia is still not well understood, but several risk factors have been identified. These include genetic factors [2], [3], nulliparity, multifetal gestations, maternal race and age, and pre-existing conditions such as preeclampsia in a prior pregnancy, chronic hypertension, kidney disease, diabetes mellitus, and obesity [1].

Numerous studies have documented an inverse association between cigarette smoking during pregnancy and preeclampsia in different populations with a reduction of risk by up to 50% [4]. The underlying mechanism for this association is still not well understood with several hypothesized pathways, including carbon monoxide-mediated inhibition of inflammation [5], enhanced vasodilation [6], suppression of platelet aggregation [7], plasminogen activation [7], apoptosis [8], [9], reactive oxygen species formation [10], and sFlt-1, an antiangiogenic factor [11], [12].

Advanced maternal age and race/ethnicity have been well documented to be significant risk factors for a number of adverse pregnancy outcomes. Gregory and Korst observed that older pregnant women experienced increased risk of a number of maternal, fetal, and placental conditions including hypertension [13]. Rates of hypertensive disorders and preeclampsia appear to vary by race and ethnicity, as do the presentation and course of the disease [13]–[23]. In the U.S., preeclampsia risk is higher in ethnic minority women compared with non-Hispanic white women, with African-American women having the highest rate [18], [22], [23]. The causal mechanisms explaining the racial and ethnic differences in hypertensive disorders during pregnancy are largely unknown. These disparities might be related to a number of risk factors that are associated with race and ethnicity for preeclampsia, including chronic hypertension, diabetes, and kidney disease [24]. For instance, it has been shown that white women are more likely to smoke heavily during pregnancy (≥20 cigarette daily) than women of other race and ethnicity [23], [25].

Despite the acknowledged disparity in hypertensive disorders during pregnancy by maternal age and race/ethnicity, few prior studies have examined the impact of their joint interactive effect with other known risk factors. The objective of this study was to examine the association between cigarette use during pregnancy and PIH by maternal race/ethnicity and age.

## Materials and Methods

This population-based, retrospective cohort study was based on the 2010 public-use U.S. natality file from Center for Disease Control and Prevention's National Center for Health Statistics. We used data based on the 1989 (unrevised) version of the birth certificate for this analysis because the 2003 release has substantial missing data for some key variables for the present study. The natality file includes data on parental demographics, medical and obstetrical characteristics and complications, and neonatal status at birth. Our study sample consisted of U.S. women who delivered singleton pregnancies between 20 and 44 weeks of gestation in 2010. Pregnancies complicated by major fetal anomalies were excluded. These exclusions resulted in a study sample of 3,113,164 pregnancies. U.S. natality data is a publicly available data set and qualified for exemption from Institutional Review Board approval.

The exposure, cigarette use during pregnancy, was indicated with a yes/no binary variable based on maternal self-report on the birth certificate. The outcome was PIH and included all women who had the condition “pregnancy-associated hypertension (i.e., PIH/preeclampsia)” or “eclampsia” checked on the 1989 version of the birth certificate [26], [27]. Pregnancy-associated hypertension was defined as pregnancy- induced hypertension after the 20^th^ week of gestation that resulted in an increase in blood pressure of at least 30 mm Hg systolic or 15 mm Hg diastolic on two measurements taken 6 hours apart [27]. Eclampsia, defined as the occurrence of convulsions and/or coma unrelated to other cerebral conditions in women with signs and symptoms of preeclampsia [27], was included in the preeclampsia group due to the small number of women affected within each ethnic group after stratification by maternal race/ethnicity and age. Mother's race and ethnicity were self-reported and categorized as non-Hispanic white, non-Hispanic black, Hispanic, non-Hispanic Asian/Pacific Islander (non-Hispanic Asian), and non-Hispanic American Indians/Alaskan natives (non-Hispanic American Indians), and Hispanics.

Factors that may be associated with maternal smoking and PIH were evaluated as potential confounders. Data for the following demographic and lifestyle variables were obtained from the birth certificate: maternal age (<20, 20 to 34, and ≥35 years), marital status (single or married), parity, Kotelchuck prenatal care index [28], gestational weight gain (<20 lbs, 20 to 39 lbs, and ≥40 lbs), and maternal medical risk factors including diabetes and chronic hypertension.

Some studies had suggested potential misclassifications of medical conditions with data from birth certificates [29], [30]. To address this issue, we attempted to validate our study findings from the natality data by replicating the same analysis as in the birth certificate data in a nationally representative sample of maternal hospital discharges from the U.S. National Inpatient Sample (NIS) database, the largest all-payer, publicly available inpatient database in the U.S. [31]. We identified in NIS records representing hospital stays for women who were pregnant or gave birth using Diagnosis Related Groups codes (765, 766, 767,768, 774, 775) assigned to inpatient visits from years 2006, 2008, and 2010. NIS data from 2006 and 2008 were included to increase the sample size due to the small number of eclampsia cases available. Multiple births were excluded (as defined by ICD9 codes V31–V37). This resulted in a NIS data sample of 12,326,151 pregnancies. We used ICD-9-CM codes to identify the exposure (i.e. cigarette use during pregnancy) and outcome (PIH) as well as maternal medical conditions as follows: tobacco use (305.1, V. 158.2), preeclampsia and pregnancy-induced hypertension (642.4×, 642.5×, 642.3×), and eclampsia (642.61, 642.62, 642.63, 642.64), diabetes (ICD9 250), and chronic hypertension (642.2, 401).

### Statistical analysis

Differences in sample characteristics by race/ethnicity were assessed by using the Pearson Chi-square test (χ^2^) for categorical variables and t-test for continuous variables. Multivariable binary logistic regression models were used to estimate the crude and adjusted odds ratios (aOR) and the corresponding 95% confidence interval (95% CI). To reduce the bias in the parameter estimation, potential confounders were included in the multivariate analysis, including maternal age, marital status, parity, Kotelchuck prenatal care index, gestational weight gain, and maternal medical risk factors (i.e., diabetes and chronic hypertension). To evaluate if maternal race/ethnicity and age are effect modifiers, the Wald test was used to test if the regression coefficient of the product term of cigarette use during pregnancy, maternal race/ethnicity, and maternal age was statistically different from zero. Advanced maternal age, defined as 35 years or older, has been associated with adverse pregnancy [32]. Therefore, we dichotomized maternal age using a cutoff value of 35. We detected a significant interaction effect (interaction term *P*- value<0.01) for the product term of cigarette use during pregnancy, maternal race/ethnicity, and maternal age. Therefore, we stratified by race/ethnicity and age in the multivariable analysis. To generate nationally representative estimates, the analyses based on the NIS sample data were weighted and adjusted for clustering data structure using the survey commands. All tests were 2 tailed and *P*<0.05 was considered significant. All statistical analyses were performed with STATA (version 10.0, STATA Corp, College Station, TX).

## Results

In this study, data from the birth certificates served as the primary source because they provide more demographic and lifestyle variables than the NIS data as potential confounders for the multivariable analysis. As expected by the differing data sources, there were statistically significant differences in selected maternal characteristics between the natality and the NIS data samples (Table 1). Women in the NIS data were slightly older than those from the natality data. Prevalence of pregnancy-induced hypertension was greater in the NIS sample than in the natality data. The prevalence of cigarette use during pregnancy, eclampsia, chronic hypertension, and diabetes were lower in the NIS data than in the natality data (Table 1).

**Table 1:** Study Sample Characteristics.

Characteristic	Natality data	NIS samples	p-value*
	(n = 3,113,164)	(n = 12,326,151)	
	%	%	
Mother's age Mean (SD)	26.6 (6.1)	27.5 (13.6)	<0.0001
Mother's age			
<20	9.4	10.1	<0.0001
20–34	76.5	75.6	
35+	14.1	14.3	
Tobacco use	9.2	2.1	<0.0001
PIH/Preeclampsia	4.1	6.7	<0.0001
Eclampsia	0.27	0.08	<0.0001
Chronic hypertension	1.3	0.7	<0.0001
Diabetes	5.0	0.8	<0.0001
Marital status	40.1	na	
Not married			
Parity		na	
1	59.0		
≥2	41.0		
Prenatal care adequacy			
Inadequate	14.7	na	
Intermediate	10.1		
Adequate	42.1		
Adequate+	33.1		
Weight gain			
0–19 lbs	24.8	na	
20–39 lbs	51.2		
40+ lbs	24.0		

Abbreviation: SD, standard deviation; NH, non-Hispanic, IH, Pregnancy induced hypertension.

*p value from Pearson Chi-square test (χ^2^) for categorical variables and t-test for continuous variables.

Table 2 shows the maternal characteristics in different ethnic groups from the natality data. All variables showed significant differences by race/ethnicity. The mean (standard deviation) maternal age of non-Hispanic Asian [30.6 years (5.3)] was higher (*P*<0.01) than that of other ethnic groups. Non-Hispanic American Indian women had the highest proportions of teenage births (16%), tobacco use during pregnancy (18.6%), PIH/preeclampsia (5.3%), eclampsia (0.6%), and inadequate prenatal care compared to women of other ethnic origins. Relative to women in other ethnic groups, non-Hispanic black women had the greatest proportions of chronic hypertension (2.8%) and being single (72.5%) whereas non-Hispanic Asian had the largest percentage of women with diabetes (8.7%).

**Table 2:** Maternal Characteristics by Race/Ethnicity, US 2010 Natality file (n = 3,113,164).

Characteristic	NH White	NH Black	NH American Indian	NH Asian/Pacific Islander	Hispanic	P Value*
	(n = 1,718,634)	(n = 402,472)	(n = 34,348)	(n = 192,141)	(n = 765,569)	
	n(%)	n(%)	n(%)	n(%)	n(%)	
Mother's age Mean (SD)	28.2 (5.8)	25.9 (6.2)	26.4 (5.8)	30.6 (5.3)	30.2 (5.4)	<0.01
Mother's age						<0.01
<20	117,061 (6.8)	62,348 (15.5)	5,503(16.0)	4,155(2.2)	104,003 (13.6)	
20–34	1,347,959(78.4)	298,208 (74.1)	26,092 (76.0)	141,816 (73.8)	567,453 (74.1)	
35+	253,614 (14.8)	41,916 (10.4)	2,753(8.0)	46,170 (24.0)	94,113 (12.3)	
Tobacco use	227,997 (13.3)	33,300 (8.3)	6,389 (18.6)	2,800(1.5)	15,931(2.1)	<0.01
						
PIH/Preeclampsia	77,812 (4.5)	20,616 (5.1)	1,835 (5.3)	4,558 (2.4)	23,505(3.1)	<0.01
Eclampsia	4,554 (0.3)	1,720 (0.4)	195(0.6)	404(0.2)	1,635(0.2)	<0.01
Chronic hypertension	21,610 (1.3)	11,330 (2.8)	601 (1.8)	1,535 (0.8)	5,363(0.7)	<0.01
Diabetes	79,167 (4.6)	17,915 (4.5)	2,397 (7.0)	16,656(8.7)	40,005 (5.2)	<0.01
Marital status						<0.01
Married	1,224,022(71.2)	110,690 (27.5)	11,519 (33.5)	162,467 (84.6)	356,277 (46.5)	
Not married	494,612 (28. 8)	291,782 (72.5)	22,829 (66.5)	29,674 (15.4)	409,292 (53.5)	
Parity						<0.01
1	741,778 (43.2)	164,284 (40.8)	12,524 (36.5)	88,947 (46.3)	269,956 (35.3)	
≥2	976,856 (56.8)	238,188 (59.2)	21,824 (63.5)	103,194 (53.7)	495,613 (64.7)	
Prenatal care Adequacy						<0.01
Inadequate	184,037 (10.7)	88,795 (22.1)	8,826 (25.7)	23,195 (12.1)	152,607 (19.3)	
Intermediate	161,565 (9.4)	43,267 (10.8)	4,719 (13.7)	21,244 (11.1)	84,207 (11.0)	
Adequate	769,893 (44.8)	144,370 (35.9)	12,315 (35.9)	84,611 (44.0)	298,586 (39.0)	
Adequate+	603,139 (35.1)	126,040 (31.3)	8,488 (24.7)	63,091 (32.8)	230,169 (30.1)	
Weight gain						<0.01
0–19 lbs	356,997 (20.8)	128,957 (32.0)	10,390 (30.3)	43,571 (22.7)	232,815 (30.4)	
20–39 lbs	899,157 (52.3)	177,754 (44.2)	15,565 (45.3)	113,758 (59.2)	388,071 (50.7)	
40+ lbs	462,480 (26.9)	95,761 (23.8)	8,393 (24.4)	34,812 (18.1)	144,683 (18.9)	

Abbreviation: SD, standard deviation; NH, non-Hispanic, IH, Pregnancy induced hypertension

*p value for the association of maternal race/ethnicity with all maternal characteristics in the study based on analysis of variance (continuous variable) or Chi-Square test (categorical variables).

Using the natality data, we found decreased odds of PIH among non-Hispanic white and non-Hispanic American Indian women who smoked during pregnancy (aOR = 0.90, 95% CI: 0.88, 0.92 and aOR = 0.80, 95% CI: 0.70, 0.91, respectively, Table 3) compared to those who did not smoke during pregnancy, after controlling for confounders that included maternal age, marital status, parity, Kotelchuck prenatal care index, gestational weight gain, chronic hypertension, and diabetes. On the contrary, non-Hispanic black, non-Hispanic Asian, and Hispanic women who smoked during pregnancy had increased likelihood of PIH, after adjusting for confounders (Table 3). The increased odds of PIH for non-Hispanic Asian women who smoked during pregnancy was also observed in the NIS sample (aOR = 1.53, 95% CI: 1.14, 2.04) after controlling for maternal age, chronic hypertension, and diabetes (Table 3). Some covariates including marital status, parity, Kotelchuck prenatal care index, gestational weight gain were not available in NIS dataset and thus could not be included in the multivariate analysis for the NIS sample.

**Table 3:** Odds Ratios for the Effect of Smoking on PIH among Ethnic Groups.

	Natality data (n = 3,113,164)				NIS data (n = 12,326,151)	
	Crude OR	95 % CI	Adjusted OR*	95 % CI	Adjusted OR+	95 % CI
NH White	0.89	0.87, 0.91	0.90	0.88, 0.92	0.99	0.94–1.03
NH Black	0.99	0.94, 1.04	1.07	1.02, 1.13	0.95	0.85, 1.06
NH American Indian	0.74	0.65, 0.84	0.80	0.70, 0.91	0.97	0.65, 1.43
NH Asian/Pacific Islander	1.73	1.44, 2.09	1.64	1.36, 1.99	1.53	1.14, 2.04
Hispanic	1.11	1.02, 1.21	1.09	1.01, 1.19	0.98	0.84, 1.14

Abbreviation: OR, odds ratio, 95% CI, 95% confidence interval.

* adjusted for maternal age, marital status, parity, kotelchuck prenatal care index, gestational weight gain, chronic hypertension, diabetes.

+ adjusted for maternal age, chronic hypertension, and diabetes.

Table 4 shows that the inverse relationship between maternal smoking and PIH was age dependent. The odds of PIH increased with age in all ethnic groups after adjusting for potential confounders. The reduced odds of PIH was only evident for non-Hispanic white and non-Hispanic American Indian women young than 35 years old who smoked during pregnancy based on the natality data. This decreased odds of PIH conferred by maternal smoking was similarly observed in non-Hispanic white women younger than 35 years old based on NIS sample, albeit with a borderline significant and weaker strength of association. It is noteworthy that the increased odds of PIH among non-Hispanic Asian women who smoked during pregnancy persisted regardless of maternal age, with stronger association among older women within this ethnic group (Table 4). Furthermore, data from both the natality and the NIS sample indicated increased odds of PIH among non-Hispanic white women 35 years or older who smoked during pregnancy compared with those who did not, with stronger association based on the NIS data (Table 4). Interestingly, non-Hispanic white women also had the lowest aOR in the older age group, suggesting that there may still be a mitigating effect of smoking within this ethnic group.

**Table 4:** Odds Ratios for the Effect of Smoking on PIH Among Ethnic Groups by Maternal Age.

	Natality data		NIS data (n = 12,326,151)	
	(n = 3,113,164)			
Women <35 yrs	Adjusted OR*	95 % CI	Adjusted OR+	95 % CI
NH White	0.89	0.86, 0.91	0.95	0.91, 1.00
NH Black	1.05	1.00, 1.09	0.91	0.81, 1.03
NH American Indian	0.76	0.66, 0.87	0.96	0.64, 1.45
NH Asian/Pacific Islander	1.66	1.35, 2.05	1.36	0.96, 1.93
Hispanic	1.09	1.00, 1.20	0.99	0.84, 1.17

	Natality data		NIS data (n = 12,326,151)	
	(n = 3,113,164)			
Women ≥35 yrs	Adjusted OR*	95 % CI	Adjusted OR+	95 % CI
NH White	1.17	1.09, 1.26	1.29	1.13, 1.47
NH Black	1.18	1.01, 1.37	1.30	0.97, 1.62
NH American Indian	1.29	0.88, 1.89	1.07	0.25, 4.63
NH Asian/Pacific Islander	1.71	1.07, 2.72	2.32	1.35, 3.99
Hispanic	1.18	0.91, 1.54	0.91	0.57, 1.46

Abbreviation: OR, odds ratio, 95% CI, 95% confidence interval.

* adjusted for maternal age, marital status, parity, kotelchuck prenatal care index, gestational weight gain, chronic hypertension, diabetes.

+ adjusted for maternal age, chronic hypertension, and diabetes.

There may be potential overlap between the 2010 US natality and the NIS sample, which includes data from year 2006, 2008, and 2010. Therefore, we conducted a subsample analysis for the 2010 US natality data set among primiparous women and the NIS sample from only 2006 and 2008, separately. In this subsample analysis, we obtained results that are largely consistent with the analysis of the US natality and the multi-year NIS sample. We observed that the association between maternal cigarette use and hypertensive disorders of pregnancy varied by maternal race/ethnicity and age (Table S1). Specifically, the decreased odds of PIH among women who smoked during pregnancy was only apparent in non-Hispanic white and American Indian women younger than 35 years old who smoked during pregnancy, based on the natality data and among non-Hispanic white and blacks only based on the NIS sample. We also observed that maternal cigarette use during pregnancy was associated with increased odds of PIH for non-Hispanic Asians younger than 35 years old based on the natality data and the increased odds was also conferred by the NIS data regardless of maternal age (Table S2).

## Discussion

Numerous studies have observed a decreased risk of both preeclampsia and gestational hypertension among women who smoked during pregnancy with an average aOR of 0.7 [4], [33]. However, to our knowledge, no prior studies have examined the joint impact of maternal race/ethnicity and age on the relationship between smoking and hypertensive disorders of pregnancy. The present study found that the association between maternal cigarette use and hypertensive disorders of pregnancy varied by maternal race/ethnicity and age. Specifically, the decreased odds of PIH among women who smoked during pregnancy was only apparent in non-Hispanic white and American Indian women younger than 35 years old who smoked during pregnancy, based on the natality data and among non-Hispanic white only based on the NIS sample. Interestingly, we also observed that maternal cigarette use during pregnancy was associated with increased odds of PIH for non-Hispanic Asians regardless of maternal age, based on the natality data and conferred for the older age group by the NIS data.

In general, we observed an association in the same direction with cigarette use during pregnancy and PIH between the two data sets, albeit with differences in the strength of association with a weaker association from the NIS data for women younger than 35 years of age, but a stronger association for mothers with age greater than or equal to 35 (Table 4). This discrepancy in findings between the two data sets may be explained by the different sources of information between the two data sets. In the NIS data, measures of interest were defined based on ICD-9 discharge diagnosis codes, whereas in the natality data smoking is self-reported by the patient and hypertensive disorders were coded by chart extractors. Hence, compared to the natality data, prevalence of maternal smoking during pregnancy in the NIS data was much lower, whereas, the prevalence of the outcome was higher (Table 1). Geller and colleagues [34] evaluated the accuracy of the ICD-9 revision codes for preeclampsia and eclampsia and observed variation in accuracy of diagnosis with a positive predictive value for severe preeclampsia of 84.8%, 45.3% for mild preeclampsia, and 41.7% for eclampsia. The potential misclassification in NIS data for exposure and the outcome were likely non-differential, however, which would bias the point estimate toward the null value and may explain the weaker strength of association observed in the younger women in the NIS data.

The inverse association between maternal smoking and PIH among non-Hispanic white and American Indian women in our study appears to be weaker compared to findings from prior research [23], [33], [35]. Misra et al. found that maternal smoking was associated with reduced odds of gestational hypertension in white women (aOR: 0.17, 95% CI: 0.12 to 0.24) but not in black women (aOR: 0.35, 95% CI: 0.09–1.37) [35]. However, results from this study may have been affected by selection bias as more than half of the eligible subjects were excluded from the study due to missing data [35]. Knuist et al. observed an inverse association between maternal smoking and preeclampsia in both white [adjusted relative risk: 0.8, 95% CI: 0.3, 1.7] and black women (adjusted relative risk: 0.5, 95% CI: 0.1, 4.4), but the results were not statistically significant. Findings from Knuist et al. may be limited by the lack of sufficient statistical power for a stratified analysis by maternal race [23].

The weaker association between maternal smoking and PIH from our study may be attributed to potential misclassification of the exposure, maternal smoking, due to recall bias or under-reporting for both natality and the NIS data. Nevertheless, this measurement error of exposure was likely non-differential, which we expect would result in the attenuation of our point estimate toward the null value [36],which may explain the weaker association in our findings. In addition, the different rates of maternal smoking and preeclampsia across ethnic groups observed by previous studies may have contributed to this discrepancy.

Our observation of the increased odds of PIH among non-Hispanic Asian women was unexpected and of particular interest. In the non-pregnant population, it has been observed that Asian and Indian adults have an increased risk of diabetes, hypertension, and dyslipidemia at lower BMI levels than European adults [37]. Additionally, in a study of ethnic variations in HELLP (hemolysis, elevated liver enzymes, low platelets) syndrome among pregnant women with preexisting hypertension, Williams et al. observed that Caucasian and Chinese women were more likely than East Indians to develop HELLP syndrome [38]. Asian women may also differ from women of other ethnic origins in socioeconomic status, stress, physical activities, diet, and other social or life style factors that could contribute to this differential risk in hypertensive disorders of pregnancy.

In general, race/ethnicity may influence health outcomes due to differences in socio-economic status, life style (i.e., diet and physical activity), access to medical care, and medical conditions among ethnic groups [19]. For instance, blacks have higher prevalence of hypertension [39] and obesity [40] than whites. Cigarette use during pregnancy was less prevalent among Asians and blacks compared to whites [35], [41], [42]. Knuistet al suggested that risk factors for preeclampsia differed in prevalence among women of different ethnic groups such that the diastolic blood pressure was the strongest predictor for preeclampsia in white women whereas high maternal age was a better predictor for preeclampsia in blacks [23]. Previous studies also suggested that the underlying pathophysiology of hypertensive disorders may be different in blacks than in whites [43], [44]. The varying rates of preeclampsia by race/ethnicity could also be attributed to genetic predisposition to developing diseases [45], [46]. Prasmusinto et al. demonstrated ethnic differences in the association of factor V Leiden mutation and the C677T methylenetetrahydrofolate reductase gene polymorphism with preeclampsia among white and Indonesian mothers [47]. Variations in the distribution in the risk factors of preeclampsia including maternal smoking among ethnic groups combined with ethnic variation in genetic polymorphisms predisposing to preeclampsia may explain the observed ethnic differences in the association between maternal smoking and preeclampsia. Further studies are needed to elucidate the biologic mechanisms underlying the ethnic differences in this relationship.

Few studies have sufficient sample size to examine the effect modifying role of maternal age in the association between maternal smoking and preeclampsia. Advanced maternal age, especially after age 35, is associated with increased risk of preeclampsia [48] and other adverse pregnancy outcomes [49]. In a large study based on birth records linked with hospital discharge data from New York City, Engel et al. found that the reduced risk of preeclampsia among women who smoked during pregnancy was limited to women aged 30 years or younger and that more advanced maternal age may be associated with greater risk of hypertension with preeclampsia [50]. Our findings are in general agreement with this observation.

Some methodological limitations of this study need to be considered in interpreting our study findings. They include the potential for inaccurate reporting, residual confounding by socioeconomic and other unmeasured maternal characteristics (e.g., stress, physical activities, nutrition), the lack of information regarding the diagnosis, timing and severity of preeclampsia, and misclassification of medical and obstetrical conditions. A prior validation study has indicated that the reporting rate of preeclampsia on birth certificates with a check-box format is fairly good, ranging from 85% to 97% when compared with risks based on hospital discharge data [51]. In our study, we used pregnancy induced hypertension to approximate preeclampsia as our outcome which may results in measurement error. It is unclear whether pregnancy induced hypertension and preeclampsia are two distinct disorders that share a similar symptom (i.e., hypertension) or if pregnancy induced hypertension is a precursor of preeclampsia [16].

Our study findings are also limited by the self-reported information on tobacco use status during pregnancy. However, the prevalence of cigarette use during pregnancy in natality data from the present study is consistent with the population-based estimate from the 2010 Pregnancy Risk Assessment and Monitoring System data from 27 states conducted by the U.S. Centers for Disease Control and Prevention [52]. England et al. reported that 24% of smokers during pregnancy were misclassified as quitters in a large multicenter randomized study of nulliparous women [53]. Llurba and associates observed a 90% concordance between self-reported smoking and cotinine levels among 125 healthy Spanish pregnant women at 24 weeks of gestation in a case control study [54]. To estimate the bias introduced by the potential misclassification of maternal smoking status in our study based on natality data file, we conducted a sensitivity analysis. Assuming a 76% sensitivity and 100% specificity of our exposure measurement, our parameter estimates remained largely the same for results shown in Table 3 and 4 after correcting for the measurement error [55]. Our study is further limited by the lack of information on the timing, intensity, and frequency of maternal smoking, which could potentially vary by race/ethnicity. In addition, our analysis used broad categorizations of ethnicity (e.g. non-Hispanic Asian and Hispanic), which may obscure an association between maternal smoking and PIH within ethnic subgroups. When analyzing different ethnic and racial groups in the present study based on a U.S. population, it is difficult to ascertain whether the categorization of maternal race/ethnicity summarizes genetics or environment. While racial group implies a specific genetic inheritance, ethnicity reflects culture and is therefore changeable. In this study no attempt has been made to distinguish between the two potential effects in the role of maternal ethnic origin as an effect modifier.

The strength of this study rests in its use of a large population-based sample of U.S. women and the availability of information on many potential confounders that may affect the risk of PIH from the natality data. The large sample size provided adequate statistical power to detect significant associations, increased precision in the risk estimates, and the ability to evaluate the potential effect modifying role of maternal race/ethnicity and age. In addition, our study used two independent data sources and observed similar findings albeit with variations in the strength of association for the effect of interest. Using the NIS data from hospital inpatient records, which is superior to birth certificate check boxes further supports and enhances the validity of our study findings.

Our study findings suggested important differences by maternal race/ethnicity and age in the association between maternal smoking and PIH. How race/ethnicity modifies this relationship is not clearly understood. It may be explained by a combination of social, behavioral, and genetic polymorphisms and disease susceptibility. While this disparity needs to be confirmed in future studies, our study results may help health professional identify specific subgroups of women who are at higher risk for PIH. Although the pathophysiologic pathways of preeclampsia are largely unknown, separation of women into etiologically homogeneous groups in future studies of preeclampsia may improve our understanding and prediction of the disease. It is plausible that women of different racial and ethnic origins may have different clinical presentations and clinical courses of preeclampsia. More research is needed to establish the biologic and social mechanisms that might explain the variations by maternal age and race/ethnicity that were observed in our study.

## Supporting Information

Table S1 **Odds Ratios for the Effect of Smoking on PIH Among Ethnic Groups.**
(DOCX)

Table S2 **Odds Ratios for the Effect of Smoking on PIH Among Ethnic Groups by Maternal Age.**
(DOCX)
