# Supplementary material for: Reassessing the Impact of Smoking on Preeclampsia/Eclampsia: Are There Age and Racial Differences?
Source: PLoS One. 2014 Oct 22;9(10):e106446. doi: 10.1371/journal.pone.0106446 (PMC4206265; doi:10.1371/journal.pone.0106446)
Supplement: Table S1 — Odds Ratios for the Effect of Smoking on PIH Among Ethnic Groups. (DOCX) [file pone.0106446.s001.docx]

Table S1. Odds Ratios for the Effect of Smoking on PIH Among Ethnic Groups

|  | 2010 Natality data among primiparous women (n= 3,113,164) | | | |  | NIS data from 2006 and 2008 (n=8,468,410) | |
| --- | --- | --- | --- | --- | --- | --- | --- |
|  | Crude OR | 95 % CI | Adjusted OR^*^ | 95 % CI |  | Adjusted OR^+^ | 95 % CI |
| NH White | 0.91 | 0.88, 0.93 | 0.89 | 0.86, 0.91 |  | 0.92 | 0.89,0.94 |
| NH Black | 1.00 | 0.92, 1.08 | 0.98 | 0.90, 1.07 |  | 0.91 | 0.85, 0.96 |
| NH American Indian | 0.78 | 0.65, 0.94 | 0.78 | 0.65, 0.95 |  | 1.10 | 0.91, 1.37 |
| NH Asian/Pacific Islander | 1.62 | 1.23, 2.13 | 1.38 | 1.03, 1.86 |  | 1.86 | 1.55, 2.23 |
| Hispanic | 1.13 | 1.00, 1.27 | 1.04 | 0.91, 1.18 |  | 0.90 | 0.83, 0.98 |

Abbreviation: OR, odds ratio, 95% CI, 95% confidence interval

^*^ adjusted for maternal age, marital status, parity, kotelchuck prenatal care index, gestational weight gain, chronic hypertension, diabetes.

^+^ adjusted for maternal age, chronic hypertension, and diabetes.
