# Supplementary material for: Reassessing the Impact of Smoking on Preeclampsia/Eclampsia: Are There Age and Racial Differences?
Source: PLoS One. 2014 Oct 22;9(10):e106446. doi: 10.1371/journal.pone.0106446 (PMC4206265; doi:10.1371/journal.pone.0106446)
Supplement: Table S2 — Odds Ratios for the Effect of Smoking on PIH Among Ethnic Groups by Maternal Age. (DOCX) [file pone.0106446.s002.docx]

Table S2. Odds Ratios for the Effect of Smoking on PIH Among Ethnic Groups by Maternal Age

|  | 2010 Natality data among primiparous women (n= 3,113,164) | |  | NIS data from 2006 and 2008 (n=8,468,410) | |
| --- | --- | --- | --- | --- | --- |
| Women < 35 yrs | Adjusted OR^*^ | 95 % CI |  | Adjusted OR^+^ | 95 % CI |
| NH White | 0.87 | 0.84, 0.90 |  | 0.90 | 0.88, 0.93 |
| NH Black | 0.97 | 0.89, 1.06 |  | 0.83 | 0.78, 0.89 |
| NH American Indian | 0.75 | 0.61, 0.91 |  | 1.07 | 0.87, 1.30 |
| NH Asian/Pacific Islander | 1.49 | 1.10, 2.02 |  | 1.64 | 1.32, 2.03 |
| Hispanic | 1.02 | 0.90, 1.17 |  | 0.91 | 0.83, 1.00 |
|  | Natality data among primiparous women (n= 3,113,164) | |  | NIS data from 2006 and 2008 (n=8,468,410) | |
| Women ≥ 35 yrs | Adjusted OR^a^ | 95 % CI |  | Adjusted OR^b^ | 95 % CI |
| NH White | 1.22 | 1.06, 1.40 |  | 1.18 | 1.10, 1.27 |
| NH Black | 1.40 | 0.91, 2.17 |  | 1.46 | 1.26, 1.70 |
| NH American Indian | 3.81 | 1.43, 10.12 |  | 1.56 | 0.76, 3.19 |
| NH Asian/Pacific Islander | 0.54 | 0.13, 2.22 |  | 2.99 | 2.14, 4.17 |
| Hispanic | 1.53 | 0.83, 2.82 |  | 0.77 | 0.58, 1.00 |

Abbreviation: OR, odds ratio, 95% CI, 95% confidence interval

^*^ adjusted for maternal age, marital status, parity, kotelchuck prenatal care index, gestational weight gain, chronic hypertension, diabetes.

^+^ adjusted for maternal age, chronic hypertension, and diabetes.
